# The Botox Paradox: How Rising Drug Costs and Reimbursement Failures Threaten Access to Botulinum Toxin Therapy in Neurology

**DOI:** 10.7759/cureus.111414

**Published:** 2026-06-24

**Authors:** Patricio S Espinosa

**Affiliations:** 1 Neurology, Espinosa Neuroscience Institute, Boca Raton Regional Hospital, Boca Raton, USA

**Keywords:** botox®, botox efficacy, botox injection, botulinum toxin type a, migraine prevention with botox, migraine treatment

## Abstract

Botulinum toxin type A (onabotulinum toxin A) remains one of the most effective therapies available for chronic migraine, cervical dystonia, blepharospasm, and spasticity disorders. Despite its established clinical efficacy, escalating acquisition costs, unavoidable medication waste, inadequate reimbursement, and increasing administrative burdens have created substantial financial challenges for independent neurology practices. The present article combines published evidence with observations from the author's private practice experience to examine the impact of these factors on treatment sustainability and patient access. The situation is particularly paradoxical because botulinum toxin remains highly profitable in aesthetic medicine while frequently operating at a loss when used to treat disabling neurological diseases. Appropriate patient selection and evidence-based utilization remain important considerations in maximizing the value of botulinum toxin therapy, particularly given clinical trial data demonstrating no superiority of onabotulinumtoxinA over placebo in patients with episodic migraine. This article examines the economic challenges facing neurology practices, the consequences for patient access, and potential reforms that could preserve the availability of this important therapy.

## Editorial

Introduction

Botulinum toxin type A has transformed the treatment of numerous neurological disorders. Since its approval for chronic migraine prevention and various movement disorders, millions of patients worldwide have benefited from reductions in pain, disability, muscle overactivity, and healthcare utilization [1,2]. Despite these clinical successes, the economics of botulinum toxin administration have become increasingly challenging for private neurology practices. Rising drug acquisition costs, medication waste, administrative overhead, restrictive reimbursement policies, and patient cost-sharing obligations have created a financial environment in which many practices struggle to continue offering treatment [3-4]. The consequences extend beyond physician practices. Reduced availability of botulinum toxin programs ultimately affects patient access, therapeutic choice, and healthcare equity [5-6]. 

High cost of treatment 

Botulinum toxin is among the most expensive medications routinely administered in outpatient neurology practice. Current acquisition costs for onabotulinumtoxin A commonly range between approximately $600 and $800 per 100-unit vial, depending on purchasing agreements and distribution channels. For chronic migraine treatment, physicians generally require two 100-unit vials to administer the PREEMPT protocol dose of 155 units every twelve weeks [1]. Consequently, approximately 45 units are discarded following each treatment session [7]. This waste is unavoidable under current packaging configurations. Unlike other medications that may be shared among patients, botulinum toxin administration is constrained by sterility, handling requirements, and regulatory standards [7]. The result is a recurring healthcare expenditure that produces no direct clinical benefit.

Minimal reimbursement margins

While insurers reimburse for botulinum toxin administration, the reimbursement margin above acquisition cost is often minimal [8]. In many circumstances, practices report reimbursement amounts only a few dollars above the actual purchase price of the medication. Margins may be insufficient to cover: prior authorization activities, insurance verification, appeals and peer-to-peer reviews, drug procurement, inventory management, storage and handling, nursing support, physician administration time, scheduling and patient communications, and billing and collections. When these operational costs are included, many independent practices effectively administer botulinum toxin at little or no profit, exacerbated by the administrative burden of prior authorizations [8-10].

Medicare and Qualified Medicare Beneficiary (QMB) challenge

The financial burden is particularly significant among Medicare beneficiaries enrolled in the Qualified Medicare Beneficiary (QMB) program. Federal regulations prohibit providers from collecting deductibles, coinsurance, or copayments from QMB beneficiaries [10-11]. Even when patients wish to contribute financially toward treatment, providers are legally prohibited from accepting payment. As a result, practices absorb the entire financial risk associated with medication acquisition and administration. This creates an unusual situation in which physicians are expected to provide high-cost specialty therapies while simultaneously being restricted from recovering portions of the treatment expense.

Cost shifting to patients

Commercial insurance plans often create different barriers. Many patients face large deductibles before coverage begins. For botulinum toxin therapy, out-of-pocket expenses can exceed $1,200 before insurance benefits become active. Consequently, financial risk is increasingly shifted from insurers to patients and physician practices. When patients cannot afford these expenses, treatment may be delayed or abandoned, highlighting disparities in healthcare access [9-18].

Chronic migraine and the PRECLUDE trial

Botulinum toxin is approved for chronic migraine prevention, defined as 15 or more headache days per month [1]. Importantly, chronic migraine represents only a minority of migraine sufferers. Population-based studies estimate that approximately 90-95% of migraine patients have episodic migraine, defined as fewer than 15 headache days monthly. Only approximately 5-10% meet the criteria for chronic migraine. The recently completed PRECLUDE Phase 3 trial further reinforces this distinction. PRECLUDE evaluated onabotulinum toxin A in patients with episodic migraine (six to 14 migraine days per month and fewer than 15 headache days monthly). Patients received either 155 units, 195 units, or a placebo. All three groups improved by approximately three migraine days per month. Neither active treatment arm demonstrated statistically significant superiority over placebo. Similarly, approximately 45-47% of patients achieved a 50% response rate across all treatment arms, including placebo [13,14]. 

These findings indicate that onabotulinum toxin A does not provide proven benefit beyond placebo in episodic migraine despite its substantial cost. Given that episodic migraine accounts for the overwhelming majority of migraine cases, the evidence-based role of botulinum toxin remains largely confined to chronic migraine [13-16].

Botulinum toxin paradox

Perhaps the greatest irony in modern headache medicine is that botulinum toxin remains highly profitable when used cosmetically but often financially unsustainable when used medically [8-11]. In aesthetic medicine, providers commonly charge approximately $10-20 per unit, generating sufficient revenue to support staffing, facility costs, and physician compensation. By contrast, neurologists are largely dependent upon insurance reimbursement systems that frequently fail to cover the true costs of treatment delivery. The same medication that generates profits in cosmetic medicine may generate losses when used to treat disabling neurological disease.

Private practice experience

Disclaimer:* The following observations are derived from the author's experience operating a private neurology practice and are presented as an illustrative example of the challenges discussed in this article. These experiences may not be representative of all practice settings.*

At our institution, maintaining a botulinum toxin program required dedicated personnel responsible for prior authorizations, inventory control, medication procurement, scheduling, billing, and patient communication. The program effectively required approximately one full-time employee devoted largely to botulinum toxin-related operations. Despite maintaining a successful clinical program, our practice experienced an estimated net loss of approximately $15,000 during 2025, attributable to botulinum toxin administration. After extensive review, we made the difficult decision to discontinue insurance-based botulinum toxin services. This decision was based entirely on economics rather than clinical efficacy. Since discontinuation, patients continue receiving care through alternative evidence-based therapies, including calcitonin gene-related peptide (CGRP)-targeted treatments, peripheral nerve blocks, infusion-based therapies, oral preventive medications, and emerging migraine treatments [16-18]. Nevertheless, the loss of botulinum toxin as a therapeutic option represents a meaningful reduction in treatment choices for patients.

As a neurologist and educator who has trained residents, fellows, advanced practice providers, and practicing physicians in the administration of botulinum toxin for multiple neurological indications, I view this trend with significant concern. The gradual disappearance of botulinum toxin programs from private practice settings threatens not only patient access but also future physician training and expertise.

Opportunities for reform

The current system creates unnecessary inefficiencies. One obvious example is medication waste. For chronic migraine, physicians administer 155 units but must typically purchase and open 200 units [1]. Manufacturers should consider: 155-unit migraine-specific vials, Smaller-dose vials for blepharospasm and hemifacial spasm. Flexible packaging options that reduce waste, Reimbursement structures recognizing unavoidable wastage, and administrative support programs for specialty practices. Such reforms could reduce healthcare expenditures while improving sustainability. Equally important, reimbursement systems should allow physician practices to earn reasonable margins when providing medically necessary specialty therapies. 

A system that rewards cosmetic use while penalizing treatment of neurological disease requires reconsideration.

Recommendations and future directions

The current system creates unnecessary inefficiencies that warrant consideration by manufacturers, payers, and policymakers. One obvious example is medication waste. For chronic migraine, physicians administer 155 units but must typically purchase and open 200 units. Manufacturers should consider developing migraine-specific vial sizes, smaller-dose vials for indications such as blepharospasm and hemifacial spasm, and more flexible packaging options that reduce unavoidable waste. In addition, reimbursement structures should better recognize medication wastage that occurs as a consequence of approved dosing protocols.

Administrative burden remains another major challenge. Prior authorization requirements, appeals, and reimbursement complexities consume substantial physician and staff resources. Streamlining authorization processes and expanding administrative support for specialty practices may improve efficiency while reducing barriers to patient care.

Finally, reimbursement models should more accurately reflect the true costs of providing specialty therapies, including medication acquisition, storage, inventory management, nursing support, and physician administration time. Sustainable reimbursement policies would help preserve patient access to medically necessary botulinum toxin therapy while supporting the viability of independent neurology practices.

Conclusion

Botulinum toxin remains one of the most valuable therapeutic tools in modern neurology. However, escalating acquisition costs, unavoidable medication waste, inadequate reimbursement, restrictive billing regulations, and increasing administrative burdens have created substantial challenges for many independent practices. These financial and operational pressures may ultimately affect patient access to evidence-based neurological treatments. Appropriate utilization of botulinum toxin according to established indications remains essential to optimizing patient outcomes and healthcare resource allocation. Ensuring continued access to this important therapy will require ongoing attention to the economic and administrative factors that influence its delivery in clinical practice.
